# Three Episodes of Neuroleptic Malignant Syndrome With Risperidone: A Case Report and Literature Review

**DOI:** 10.1155/crps/6669246

**Published:** 2025-09-11

**Authors:** Forouzan Elyasi, Solmaz Alaei, Fatemeh Heydari, Mehran Zarghami

**Affiliations:** ^1^Sexual and Reproductive Health Research Center, Mazandaran University of Medical Sciences, Sari, Mazandaran, Iran; ^2^Psychiatry and Behavioral Sciences Research Center, Addiction Institute, Mazandaran University of Medical Sciences, Sari, Mazandaran, Iran; ^3^Department of Psychiatry, Faculty of Medicine, Mazandaran University of Medical Sciences, Sari, Mazandaran, Iran; ^4^Department of Psychiatry, Faculty of Medicine, Hamedan University of Medical Sciences, Hamedan, Iran; ^5^Department of Anesthesiology and Critical Care Medicine, Faculty of Medicine, Mazandaran University of Medical Sciences, Sari, Mazandaran, Iran

**Keywords:** neuroleptic malignant syndrome, recurrent, rhabdomyolysis, risperidone

## Abstract

**Background:** Neuroleptic malignant syndrome (NMS) is an idiosyncratic and life-threatening side effect that usually occurs in response to dopamine receptor antagonist medications. Despite increased awareness, the diagnosis of NMS remains challenging due to its wide differential diagnoses, which can lead to delayed treatment and increased mortality or premature reinitiation of the causative agent, culminating in recurrent NMS, a phenomenon with limited reports. This case presents a patient who experienced three episodes of NMS within 1 year, all triggered by risperidone.

**Case Presentation:** A 58-year-old male patient with schizophrenia presented to the emergency department of a university hospital in Northern Iran, due to decreased consciousness, fever, and rigidity. Initial laboratory results showed elevated creatine phosphokinase (CPK) at 14,949 U/L. He had two previous episodes of rhabdomyolysis and hospitalization in the past year. Review of prior hospital records indicated treatment for rhabdomyolysis with symptoms consistent with NMS, without making this diagnosis.

**Conclusion:** In any patient treated with dopaminergic drugs who suffer from mental status changes, muscle stiffness, high fever, and dysautonomia, especially who have complications such as rhabdomyolysis, kidney failure, seizures, leukocytosis, and increased CPK and lactate dehydrogenase (LDH), the possibility of NMS should be considered.

## 1. Introduction

Neuroleptic malignant syndrome (NMS) is a rare, acute, idiosyncratic, and life-threatening complication associated mainly with the use of antidopaminergic drugs, including antipsychotic agents [1]. The clinical manifestations of NMS include hyperthermia, rigidity, tremor, autonomic instability, diaphoresis, dysphagia, altered mental status, leukocytosis, and elevated creatine phosphokinase (CPK) levels [2]. Recent studies have indicated a decline in the incidence of NMS. This reduction is attributed to the increased use of atypical antipsychotics compared to typical ones, resulting in a current incidence rate of 0.01%–0.02% among patients treated with antipsychotics [3]. Historically, the mortality rate associated with NMS was estimated to exceed 30%. However, with improved physicians' awareness and the introduction of second-generation antipsychotics (SGAs), the mortality rate has significantly decreased to approximately 10% [4]. Despite increased awareness, the diagnosis of NMS remains challenging due to its wide differential diagnoses [4], which can lead to delayed treatment and increased mortality or premature reinitiating of the causative agent, culminating in recurrent NMS, a phenomenon with limited reports.

The first documented case of recurrent NMS was reported in 1984. This case involved a patient with schizophrenia and borderline intelligence. The patient experienced multiple episodes of NMS following antipsychotic medication [5]. Since then, several cases of recurrent NMS have been reported in the scientific literature, though this phenomenon is still considered relatively rare. In 1986, a case was documented involving a patient with schizophrenia treated with two antipsychotics who experienced recurrent episodes of NMS accompanied by hyponatremia following repeated use of his previous medications [6]. Also, a 42-year-old woman was reported who had developed NMS three times with three different antipsychotics [7]. This report presents a patient who experienced three episodes of NMS within 1 year, all triggered by the same antipsychotic agent.

## 2. Case Presentation

A 58-year-old male patient with schizophrenia presented to the emergency department of a university hospital in Northern Iran in February 2020 with decreased consciousness, fever, and muscle rigidity. The patient was intubated due to aspiration pneumonia. A chest CT scan revealed pulmonary involvement and lobar lesions, predominantly in the lung bases. A brain CT scan showed no pathological findings. Brain CT scans in patients with NMS often reveal no significant pathological findings, effectively ruling out cerebral edema, hemorrhage, or mass lesions as potential causes of altered mental status. His medication regimen included risperidone 2 mg daily, lorazepam 0.5 mg nightly, and omeprazole 20 mg each morning.

**Figure 1:**
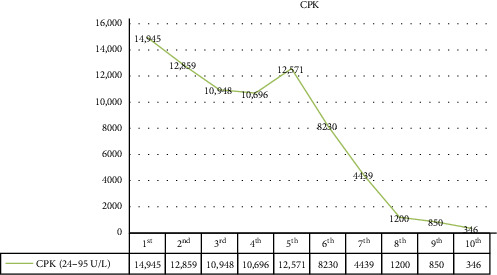
Laboratory data: creatine phosphokinase.

The patient received treatment for aspiration pneumonia and rhabdomyolysis with intravenous meropenem, vancomycin, ciprofloxacin, and enoxaparin. On the first day of hospitalization, arterial blood gas analysis revealed a pH of 7.47, HCO_3_^−^ at 18.9 mmol/L, and *P*_CO_2__ at 26 mmHg. Initial laboratory results showed elevated CPK at 14,949 U/L, hyponatremia (119 mmol/L), leukocytosis, abnormal liver function tests, and hematuria in urinalysis (Figure 1).

On the first day of admission, a psychiatric consultation resulted in a request for urine toxicology screening. On the second day, a hospital liaison psychiatrist evaluated the patient. Based on paraclinical findings, generalized rigidity, and autonomic symptom fluctuations observed during the examination, the patient was diagnosed with NMS according to DSM-5 criteria. The differential diagnosis of NMS includes conditions with similar clinical presentations, such as central nervous system infections—particularly meningitis and encephalitis. However, in the present case, these possibilities were excluded based on normal neuroimaging results and the absence of meningeal signs. The patient's clinical picture, its temporal association with risperidone use, and characteristic laboratory findings (including elevated CPK levels) supported the diagnosis of NMS over infectious causes. Bromocriptine 2.5 mg three times daily was prescribed. Due to its unavailability in the hospital, amantadine 100 mg three times daily and lorazepam 2.5 mg daily in divided doses were initiated.

A telephone consultation with the patient's treating psychiatrist at the care facility revealed two previous episodes of rhabdomyolysis and hospitalization in the past year, first in April 2019 and again in November 2019. Review of prior hospital records indicated treatment for rhabdomyolysis with symptoms consistent with NMS. During the first admission, a psychiatric resident noted the patient's noncooperation in interviews, a fixed stare, rigidity of the upper and lower extremities, and a CPK level of 8213 U/L, recommending the discontinuation of risperidone (10 mg daily) and initiation of lorazepam.

During the second admission, CT scans showed patchy ground-glass opacities in both lung fields and hyponatremia (sodium 115 mEq/L). The patient's CPK was 9327 U/L and lactate dehydrogenase (LDH) was 1383 IU/L. The patient improved with supportive treatment, rhabdomyolysis management, and discontinuation of risperidone during both hospitalizations. However, due to a lack of NMS diagnosis at discharge, risperidone was reinitiated postdischarge, leading to recurrent NMS episodes.

**Figure 2:**
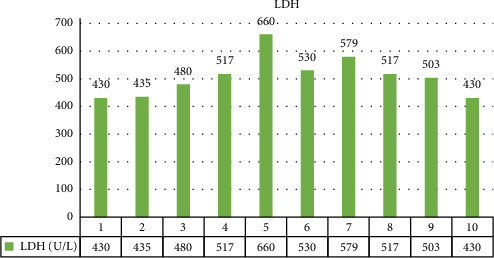
Laboratory data: lactate dehydrogenase.

**Figure 3:**
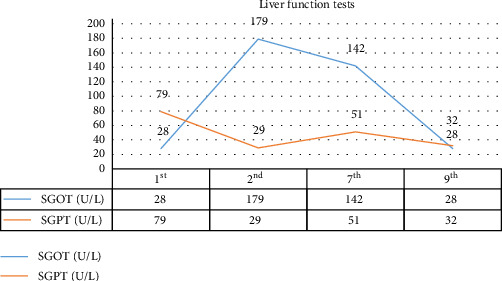
Laboratory data: serum glutamic-oxaloacetic transaminase (SGOT) and serum glutamic pyruvic transaminase (SGPT) levels.

**Figure 4:**
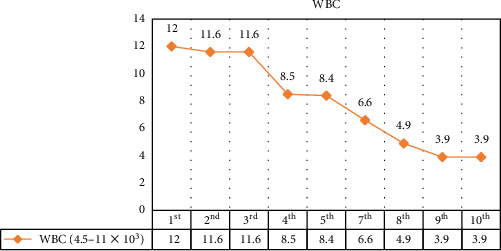
Laboratory data: patient's white blood cells.

The changes in CPK, LDH, serum glutamic-oxaloacetic transaminase (SGOT), and serum glutamic pyruvic transaminase (SGPT) levels are illustrated in Figures 1–4.

The patient was discharged 10 days after the most recent admission with improved consciousness, resolved hyponatremia, and resolved both aspiration pneumonia and NMS symptoms. It was recommended that risperidone should never be prescribed again.

## 3. Discussion

This report describes a patient who experienced NMS three times with risperidone, in which the treatment of the first two episodes was limited to the treatment of rhabdomyolysis and aspiration pneumonia, and risperidone administration continued regardless of the cause of these symptoms.

One of the early reports in 1986 described a patient treated with flupenthixol decanoate (80 mg every 2 weeks), along with trifluoperazine (20 mg daily) and orphenadrine (100 mg daily) [6]. The use of high-potency neuroleptics and represcription before full recovery from the initial NMS episode are recognized as significant risk factors for recurrence of the syndrome [8]. There are limited reports of recurrent NMS. The characteristics of these case reports, including the year of occurrence, number of recurrences, clinical features of the syndrome, the causative neuroleptic drug, other prescribed medications, and so on, are summarized in Table 1.

In 1988, Susman and Addonizio [8] reported four cases of bipolar disorder that were treated with lithium and classic first-generation antipsychotics and developed recurrent NMS. They stated some were not “independent recurrences,” but “exacerbation of episodes that had begun to wane secondary to reintroducing neuroleptics.” They discussed that safe restarting antipsychotics shortly after an episode of NMS is possible. However, they acknowledged that the syndrome could recur, possibly after a considerably longer interval. Besides, their unexpected finding was that mild recurrences may be self-limiting. In their literature review, research evidence suggests that the risk of recurrence increases in the presence of predisposing factors such as agitation or metabolic disturbances. Mood disorders, especially mania, increase the risk of NMS development too. However, it is unclear whether mania increases the risk of NMS or the agitation, dehydration, and fatigue associated with mania. Besides, the dosage of of antipsychotic medication, its relative potency, the simultaneous administration of lithium, and the cessation of dopaminergic drugs are likely to have a direct correlation with the risk of developing NMS and its potential recurrence.

Finally, they recommended administering low potency antipsychotics and avoiding the coadministration of lithium to reduce the risk of NMS recurrence [8].

In 1992, Bambrick and Wilson [14] reported recurrent NMS in a 29-year-old male with mild intellectual disability and psychotic disorder. He was treated with haloperidol 5 mg thrice daily, carbamazepine 200 mg twice daily, and orphenadrine 100 mg twice daily. After the NMS episodes, due to worsening psychotic symptoms and aggression, sulpiride 200 mg was started, with close monitoring. One week later, the dose of sulpiride was increased to 400 mg daily; 3 days later, signs and symptoms of NMS were observed. He has had no further antipsychotic and no further NMS [14].

Another case from 1989 involved a 75-year-old delusional patient with dementia who was treated with amantadine after developing NMS from a low dose of haloperidol (0.5 mg three times daily) and experienced a recurrence of the syndrome following the discontinuation of amantadine [11]. In 1992, Boyd [15] reported a 38-year-old woman with mild intellectual disability who developed NMS following the unintended reintroduction of an antipsychotic.

Hamidi et al. [19] reported a 56-year-old patient with schizophrenia in 2010 who developed three episodes of NMS after the age of 50. The patient was under treatment with risperidone plus clozapine in the first episode and then, was prescribed only clozapine during the second episode. However, no neuroleptic was administered during the 45-month interval between the second and third fatal episodes of NMS; the patient was managed only with electroconvulsive therapy (ECT) and as-needed promethazine. The patient consistently exhibited subsyndromal catatonic features during this period. Several reports have suggested a potential genetic predisposition to developing NMS [19]. They suggested that this could indicate a “trait vulnerability” to NMS, which requires the presence of a “state variable"—such as a medication—for the full manifestation of the syndrome [25].

In 2013, González-Blanco et al. [22] reported an-81-year-old woman suffering from major depressive disorder with psychotic features who developed NMS 4 days after the administration of a maximum dose of 4 mg/day haloperidol. After recovering with discontinuation and supportive treatment of this syndrome, she had NMS again 10 days after the administration of 2.5 mg/day risperidone, as well as parenteral metoclopramide due to vomiting. Atrial fibrillation, coronary syndrome, pneumonia, bacteremia due to methicillin- and aminoglycoside-resistant *Staphylococcus aureus*, and splenic infarction complicated the clinical picture. Eventually, the patient improved with eight sessions of ECT [22].

In the same year, Ouyang and Chu [20] reported a 46-year-old woman with schizophrenia who had developed fever, tachycardia, disorganization, altered consciousness, muscle stiffness, bradykinesia, and urinary retention only 7 days after treatment with 10 mg intramuscular haloperidol and 10 mg oral aripiprazole twice daily. She had leukocytosis and increased CPK and liver enzymes. With the discontinuation of neuroleptic drugs and supportive therapies, these symptoms disappeared. But 4 years later, while her psychotic symptoms had subsided on low-dose clozapine and amisulpride and then only 50 mg clozapine as monotherapy, following a family stressor and exacerbation of psychotic symptoms and prescribing a 5-day course of intramuscular haloperidol and then increasing the dose of clozapine to 200 mg in 3 days, she suffered NMS again. Once again, by stopping clozapine and supportive treatments, and of course, after bromocriptine administration, the symptoms of this syndrome subsided. Then, the patient was treated with quetiapine, and there was no problem in the 1-year follow-up [20].

In 2015, Mahendran et.al. [18] reported a 39-year-old man with schizophrenia who developed five recurrences of NMS between 2 weeks and 3 years after the prescription of first-generation antipsychotic and SGA. The neuroleptics he used in each episode were: chlorpromazine (400 mg/day) plus fluphenazine decanoate (18.75 mg monthly), trifluoperazine (15 mg/day), thioridazine (its dosage is not reported), promazine (50 mg/day), clozapine (200 mg/day), and risperidone (4 mg/day). Finally, he was prescribed quetiapine 400 mg/day, which was more stable at 6-month follow-up [18].

In 2018, Verma et al. [23] highlighted the diagnostic and management challenges of recurrent NMS in a general medical service. The patient, a 69-year-old male with schizophrenia, was transferred from a psychiatric hospital to a general hospital. He experienced NMS recurrence 3 weeks after restarting antipsychotics, including haloperidol 35 mg daily and chlorpromazine 25 mg daily. Following this episode, clozapine was introduced after a 3-week drug-free period and no further recurrences were noted [23].

In 2023, a 19-year-old male with somatic symptom disorder was reported to have severe recurrent NMS just 2 days after recovery from a previous episode. He was on atypical antipsychotics, including olanzapine 20 mg daily, quetiapine 300 mg daily, and levomepromazine 150 mg daily, but had poor compliance. The recurrence occurred 2 days after antipsychotic reinitiation due to exacerbated psychiatric symptoms postdiscontinuation. During the second episode, the patient presented severe symptoms, including rhabdomyolysis, acute renal failure, and disseminated intravascular coagulation. To manage agitation, dexmedetomidine was administered for 2 weeks without antipsychotics [24]. Maeda et al. [24] emphasized that a short interval between the reinitiation of antipsychotics following NMS carries two significant risks: it not only increases the likelihood of recurrence but also exacerbates the severity of subsequent NMS episodes. In our patient, due to failure to diagnose NMS at discharge, risperidone was restarted after release, leading to a recurrence of NMS. Digitizing medical records and accurately recording the details of the procedures performed on patients may reduce the likelihood of such errors. However, despite a reduction in the risperidone dose (9 mg/day on the first admission to 2 mg/day on the third admission), CPK levels gradually increased with each admission. This suggests that the risk of relapse may be related to the time interval between resolution of NMS and reinitiation of antipsychotic medication. However, both the incidence of recurrent NMS in the literature and clear guidelines on the optimal timing for restarting antipsychotics are limited. While it is generally recommended to wait at least 2 weeks before reinitiating antipsychotic therapy, this interval has not been explicitly defined in most guidelines [4, 22, 26, 27].

When reinitiating antipsychotics, low-potency or atypical antipsychotics at minimal doses are recommended to reduce the mortality and hazard of repeated NMS [22]. Some articles suggest a minimum 5-day interval post-NMS resolution before resuming antipsychotic therapy, with close monitoring after reinitiation [10]. Moreover, Kaur et al. [28] stated that interacting medications may contribute to the pathogenesis of NMS, and that the syndrome can occur even at very low doses of antipsychotic drugs and after long-term treatment. This highlights the unpredictable nature of this clinical condition [28]. In 2018, Elyasi et al. [29] reported a patient with schizoaffective disorder treated with risperidone and clozapine who developed NMS with ileus as an autonomic symptom. Due to severe agitation from recurrent mania and psychosis during NMS, the patient was treated with bromocriptine and ECT. Both symptoms were controlled, and weekly ECT sessions were prescribed for 5 weeks as preventive treatment to extend the interval between NMS resolution and restarting of antipsychotics [29].

In addition to idiosyncratic genetic vulnerability [25], previous investigations have implied presence of delirium [30], high environmental temperature [31], high parenteral doses of neuroleptics [32], and even abrupt withdrawal of antipsychotic and dopaminergic agents, such as antiparkinsonian medications [33, 34] as factors that may increase the possibility of NMS.

Another point to note is that neither is the pathogenesis of this syndrome fully understood [7], nor are there uniform criteria for the diagnosis of NMS. The symptoms of this syndrome are so variable and diverse and can be so complicated by other problems that further and more detailed clinical research is needed [35]. Although the classic syndrome is characterized by altered mental status, muscle stiffness, and fever, its presentation can be quite heterogeneous [36]. Many criteria show shortcomings and identify too many false positive or false negative cases due to their definition [35]. In this regard, a validation study assessing the international experts consensus diagnostic criteria, which incorporates priority points based on the significance of each criterion for diagnosing NMS, determined that the criterion of “severe” muscle stiffness may be more restrictive than the standards typically employed by experienced clinicians in practice [36]. On the other hand, no laboratory test result definitively diagnoses NMS. Nevertheless, specific laboratory investigations may be necessary to evaluate the severity and complications of the condition or to exclude other potential diagnoses [37]. For this reason, since the introduction of NMS, the idea of different varieties of NMS has been proposed, and some researchers have proposed atypical types of NMS [38, 39].

On the other hand, other conditions, disorders, and diseases that have manifestations similar to NMS should always be considered; from more benign drug-induced extrapyramidal symptoms to lethal catatonia and malignant hyperthermia, as well as amphetamines and cocaine use side effects, serotonin syndrome, agitated delirium, heat stroke, toxic encephalopathies, central nervous system infections, and status epilepticus [40–46].

These are the cases that increase the possibility of nondiagnosis and, as a result, the recurrence of NMS.

## 4. Conclusion

In any patient treated with antidopaminergic agents who presents with mental status changes, muscle rigidity, high fever, and signs of dysautonomia—especially those with complications such as arrhythmia, rhabdomyolysis, hyperkalemia, renal failure, seizures, leukocytosis, and elevated CPK and LDH levels—the possibility of NMS should be strongly considered. Given the risk of NMS recurrence, if reintroduction of dopaminergic agents becomes necessary after symptom resolution, extreme caution is essential. The medication should be reintroduced at low doses, preferably using low-potency atypical neuroleptics, after a minimum interval of 15 days, with regular monitoring of CPK levels.

Recurrent NMS remains a rare but significant clinical challenge, especially in patients with a history of neuroleptic treatment. Clinical vigilance, continuous monitoring, and cautious reinitiation of therapy are key elements in the effectively managing of these patients.

## Figures and Tables

**Table 1:** Case reports of recurrent neuroleptic malignant syndrome.

	Authors	Country/race	Year	Age	Gender	How many times	Antipsychotics first episode	Antipsychotics second episode	Antipsychotics third and more episode	Other medications	Medical comorbidity	Psychiatric disorder
1	Hermesh et al. [5]	USA Patient: Persian	1984	–	Woman	2	Tiapride 100 mg two times daily	Haloperidol	–^a^	–	–	Schizophrenia borderline intelligence
2	Lavie et al. [7]	USA South Louisiana	1985	42	Woman	3	Trifluoprazine 1 mg three times a day	Chlorpromazine 300 mg	Haloperidol oral dose 2 mg	First episode: benztropine 1 mg three times a day, hydrochlorotiazide 25 mg once daily Second episode: trihexylphenidyl 2 mg, desipramine 100 mg, cholorpropramide 500 mg	–	History of psychiatric illness
3	Gibb et al. [6]	UK	1986	44	Man	2	Flupenthixol decanoate 80 mg fortnightly, trifluoperazine 20 mg, and orphenadrine 100 mg daily	Flupenthixol decanoate 80 mg fortnightly, trifluoperazine 20 mg, and orphenadrine 100 mg daily	–	–	Hyponatremia	Paranoid schizophrenia (start 1958)
4	McCarthy et al. [9]	Irland	1988	36	Man	2	Chlorpromazine (200 mg, q.d.s.) depot flupenthixol	Flupenthixol (80 mg, i.m., fortnightly), trifluoperazine(20 mg, twice a day) chlorpromazine (200 mg, nocte)	—	Second episode: benztropine (2 mg, mane) amylobarbitone sodium (200 mg, q.d.s.),	Craniotomy 15 years previously	Paranoid schizophrenia
5	Wells et al. [10]	USA	1988	45	Man	3	Thiothixene 60 mg/day po for several years- thiothixene was discontinued. On Day 4 of hospitalization, thiothixene was restarted at 15 mg/day	Single dose of mesoridazine 300 mg im	Thioridazine 200 mg/day was initiated . The dosage was increased to 300 mg/day	Third episode: lithium and carbamazepine	—	Mild mental retardation
6	Hermesh et al. [11]	Israel	1989	75	Man	2	Haloperidol at a dose of 0.5 mg, three times a day	Amantadine withdrawal	—	—	Mild untreated Parkinson's disease for the last 3 years	Senile dementia with delusional features and very aggressive behavior
7	Slack and Stoudemire [12]	USA	1989	18	Man	2	Oral trifluoperazine (20 mg/day) and lithium carbonate	Thioridazine 50 mg/day	—	Lithium	—	Mildly mentally retarded Intermittent manic psychotic episodes
8	Lazarus et al.[13]	USA	1991	31	Man	2	Thioridazine 100 mg three times a day	Fluphenazine 5 mg three times daily	—	—	Elevated urinary VMA and catecholamines Hepatitis B surface antibody positive and surface antigen negative	Mental retardation Inv dup(15)^a^
9	Bambrick and Wilson [14]	England	1992	29	Man	2	Haloperidol (5 mg tds)	Sulpiride 400 mg	—	Carbamazepine (800 mg, twice a day) and orphenadrine (100 mg, twice a day)	—	Mild mental handicap and a superadded psychotic disorder
10	Boyd [15]	USA Caucasian	1992	38	Woman	—	—	—	—	—	—	Mild mental retardation
11	Gheorghiu et al. [16]	Israel	1999	70	Woman	3	Chlorprothixene	Zuclopenthixol HCl	Olanzapine	—	—	Schizoaffective disorder
12	Askin et al. [17]	—	2000	—	—	—	Haloperidol	—	—	—	—	—
13	Mahendran et al. [18]	—	2000	39	Man	5	Depot injection of fluphenazine decanoate, 18.75 mg monthly, chlorpromazine 400 mg/day	Trifluoperazine which was increased to 15 mg/day	3rd: Thioridazine 4^th^: clozapine 5^th^: risperidone, which was increased from 1 to 4 mg/day	First episode: benzhexol 6 mg/day and diazepam 10 mg/day	—	Schizophrenia
14	Hamidi et al. [19]	Iran	2010	56 (in 36, 52, 56)	Man	3	Risperidone and clozapine	400 mg/day of clozapine and risperidone	—	Lithium (before the first and second episode)	—	Schizophrenia
15	Ouyang and Chu [20]	China	2013	46	Woman	2	Haloperidol and aripiprazole intramuscular haloperidol 10 mg bid and oral aripiprazole 10 mg bid	Haloperidol and clozapine intramuscular haloperidol 5 mg bid	—	—	—	Schizophrenia
16	Weber et al.[21]	Germany	2005	75	Woman	—	Recurrent spontaneous “neuroleptic malignant syndrome” in the absence of neuroleptic medication-NMS-like condition in severe degenerative dementia	—	—	—	—	Probable dementia with Lewy bodies
17	González-Blanco et al. [22]	Asturias (HUCA)	2013	81	Woman	–	Haloperidol maximum dose of 4 mg/day	Risperidone 2.5 mg/day metoclopramide	—	Venlafaxine, lorazepam, indapamide, and pantoprazole	Hypertension, cavernous hemangioma of the liver and bilateral gonarthrosis	Severe depressive episode with psychotic symptoms
18	Verma et al. [23]	Caucasian	2018	69	Man	–	—	Fuphenazine switched to haloperidol 35 mg per day	—	—	—	Schizophrenia
19	Maeda et al. [24]	Hiroshima, JPN	2023	19	Man	2	Olanzapine 20 mg daily, quetiapine 300 mg daily, and levomepromazine 150 mg daily	Olanzapine 20 mg and quetiapine 300 mg daily	—	—	—	Somatic symptom disorder

*Note:* “–”: not reported in the article.

^a^Inverted duplication of chromosome 15, a known genetic abnormality linked with developmental delay and neurological symptoms.

## Data Availability

The data that support the findings of this study are available from the corresponding author upon reasonable request.

## Nomenclature

CPK: Creatine phosphokinase
ECT: Electroconvulsive therapy
ED: Emergency department
NMS: Neuroleptic malignant syndrome
SGAs: Second-generation antipsychotics.
